# Gender and addiction and other mental disorders comorbidity: sociodemographic, clinical, and treatment differences

**DOI:** 10.1007/s00737-023-01353-w

**Published:** 2023-08-04

**Authors:** Silvia Díaz Fernández, Juan José Fernandez Miranda, Francisco Pascual Pastor, Francisco López Muñoz

**Affiliations:** 1 Asturian Mental Health Service Area V- Hospital Univ. Cabueñes, Servicio de Salud del Principado de Asturias (SESPA), Gijón, Spain; 2 Asturian Institute on Health Research (Instituto para la Investigación Sanitaria del P° de Asturias-ISPA), Oviedo, Spain; 3 Unidad de conductas adictivas, Servicio Valenciano de Salud (SVS), Alcoi, Spain; 4 PREVENGO, University Miguel Hernández, Elche, Spain; 5 Faculty of Health Sciences, University Camilo José Cela, Madrid, Spain; 6 Neuropsychopharmacology Unit, Hospital 12 de Octubre Research Institute, Madrid, Spain

**Keywords:** Gender, Comorbidity, Substance use disorder, Mental disorder, Prevalence, Treatment

## Abstract

The co-occurrence of substance use disorders (SUD) and other mental disorders (OMD) is assumed to be high, but the details are uncertain in Spain. The objective of the present study was to know the prevalence of this comorbidity, as well as the pharmacological treatment, both in specific addiction treatment networks and in mental health networks, with a gender perspective. Observational, multicenter study, with a randomized sample, of patients under treatment for SUD or OMD in Spain (*N* = 1783). A specific questionnaire, collecting sociodemographic and clinical variables, diagnosed SUD and OMD, and prescribed psychotropic drugs, was completed by treating clinicians. Differences between females and males were searched. A high prevalence of OMD was found in those patients treated for their SUD (71%), and also of diagnoses of SUD (59%) in people treated for OMD. Significant relationships between addiction to certain substances and specific mental disorders were found (with no main differences between women and men). The treatments for OMD were very common in the addiction treatment networks, but that of SUDs in those patients treated in the mental health networks was less than expected. A high prescription of benzodiazepines was found. Women were less frequently diagnosed with cannabis, opioid, and especially cocaine use disorders, and they had fewer psychotic disorders and more affective, anxiety, sleep, and eating disorders, with the rest being the same, including personality disorders. Women had fewer treatments with agonists and more with antagonists, and more prescriptions of anxiolytics and antidepressants. This study provides preliminary information on the coexistence in routine clinical practice of addictive disorders and other mental disorders in Spain, and on the treatment provided, and shows differences in prevalence and clinical characteristics, and especially in treatment approaches between women and men. Thus, should be useful to adapt the treatment response with greater precision, and with a gender perspective.

## Introduction

There is a high prevalence of co-occurrence between substance use disorder (SUD) and other mental disorders (OMD) (Pascual-Pastor et al. 2017; Hunt et al. 2018). Among the general population, the odds ratio of suffering from a psychiatric disorder among substance users is higher, at around 3 or 4, than among the non-using population (Torrens et al. 2015; Hasin and Grant 2015; Pascual-Pastor et al. 2017). More than a third of people diagnosed with a mental disorder abuse or are addicted to psychoactive substances (Arias et al. 2016; Hunt et al. 2018). The prevalence differs between the general population and the population undergoing treatment, being higher in the latter (Torrens et al. 2015). The importance of this comorbidity is also due to the severity of clinical presentation and social issues, the difficulties in tackling, and it and its association with worse treatment results for those affected (Torrens et al. 2015; Priester et al. 2016; Pascual-Pastor et al. 2017; Daigre et al. 2017; Spivak et al. 2020). However, the details of substance use disorders and other mental disorders comorbidity are uncertain in Spain (Gual 2007; Roncero et al. 2011; Arias et al. 2016).

Moreover, the proper use of medication is an important element in the treatment of patients with comorbidity since it significantly affects the stabilization of both psychiatric and addictive symptoms, thereby helping to increase the effectiveness of other treatments, such as the psychosocial intervention (Iqbal et al. 2019; Arias-Horcajadas et al. 2020).

In recent years, clear pharmacological measures have been discussed with regard to the specific co-occurrence of SUD and other mental disorders such as psychotic (Azorin et al. 2016; Crockford and Addington 2017; Werner and Covenas 2018), affective (Tirado Muñoz et al. 2017; Salloum and Brown 2017; Hillemacher and Frieling 2019), and anxiety disorders (Smith and Randall 2012; Sáiz Martínez et al. 2014). The best outcomes are achieved with combined use of medications and addiction-based psychosocial interventions (Crockford and Addington 2017), although there is a very limited number of effective pharmacotherapy (Smith and Randall 2012; Hillemacher and Frieling 2019), and an even smaller number of psychosocial interventions (Salloum and Brown 2017).

Similar measures and insufficient evidence also exist to address the specific substance causing the disorder together with other comorbid mental disorders, whether legal, such as alcohol and anxiolytics (Florez-Menendez et al. 2018; Guardia-Serecigni and Flórez-Menéndez 2018; Vitali et al. 2018; Hillemacher and Frieling 2019), or illicit, such as cocaine (Álvarez et al. 2013; Ochoa-Mangado et al. 2018) or opioids (Maremmani et al. 2013; Fernández-Miranda et al. 2019). Females suffer from psychiatric comorbidity, mainly depression and PTSD, more frequently than males, and treatment response is also different for women and men: women show a greater risk for relapse. These differences could be explained in part by biological differences between both sexes, and in part due to that women experience more stigma, and a high prevalence of intimate partner violence, which, in turn, led to more risk for psychiatric comorbidity (Thibaut 2018; Tirado-Muñoz et al. 2018; Fonseca et al. 2021)..

Depressive symptoms would be related to the treatment outcomes since they could be associated with higher craving and consumption relapse in women than in men (Torrens et al. 2011; Erol and Karpyak 2015). On the other hand, greater severity of the depressive episode seems to implicate lower compliance with the treatment (Gjestad et al. 2011) and a lower response rate to it (Merrill et al. 2014).

In general, women face more barriers to access to services if they have addictive disorders, including higher stigma (Thibaut 2018; Fonseca et al. 2021). No clear differences have been described in terms of pharmacological treatments response in alcohol or opioid addiction (McHugh et al. 2013). Treatment retention has not shown differences in men and women (Korte et al. 2011), maybe except for depression and alcohol use disorder (Gjestad et al. 2011). In treating comorbidity, some studies show better results in attracting women with addiction for treatment and higher adherence rates with differentiated treatments for women (Ashley et al. 2003; Coughey et al. 1998).To learn more about the prevalence and the pharmacological treatments provided for the co-occurrence of SUD with other mental disorders in Spain, with a gender perspective, a study was designed with patients treated in both the mental health and the addiction networks of the different autonomous communities (regions). The specific aim of the study was to determine the differences between males and females regarded to sociodemographic, clinical, dual diagnostic, and pharmacological treatments received for both disorders in samples of mental health and specific addiction networks. Since the aim is to provide an overall picture of each of these treatment networks, the design tries to ensure that the sample’s composition reaches the highest representativeness of the universe from which it is drawn.

## Method

An observational, cross-sectional, multicentre study, with a random sample of patients undergoing treatment for addictive disorders or other mental disorders throughout Spain (*N* = 1783) was carried out. A questionnaire, specifically designed by the study authors (Appendix), on patients’ diagnoses and treatments in specific addiction and mental health treatment networks (detoxification units, outpatient programs, hospitals/day centers, acute psychiatric units, therapeutic communities, medium-stay and rehabilitation units, mental health units/centers, prisons) was completed by health professionals (physicians or psychologists) working in those settings, always guaranteeing highest levels of confidentiality. The patients were anonymized from the start.

The study population was people receiving treatment in mental health or addiction care networks in Spain who were aged over 18 years and had a diagnosis of substance use or other mental disorder at the time of the survey. The diagnoses were performed following the ICD-10 or DSM-5 criteria and were already in their medical records or were made by the professionals treating them and participating in the study. Non-probabilistic, convenience, and consecutive sampling was used. Sample bias was reduced by conducting the survey on the same day each week for four consecutive weeks on all patients attending the professional’s office that day (considering it this way as a randomized sampling).

The variables studied were sociodemographic (age, sex/gender, employment status, living arrangements), related to somatic pathologies (HBV, HCV, HIV), substance use disorders (ICD 10: F10–F19), other mental disorders (ICD 10: F00–F09 and F20–F99), and psychopharmaceuticals prescribed both for the SUD (opioid agonists/opioid antagonists/disulfiram) and for the OMD (antipsychotics/mood stabilizers/antidepressants), and anxiolytics (considered treatments in both groups). Data collection was carried out in fifteen of the seventeen regions of Spain. Differences between male/female were searched.

Descriptive and inferential statistics were performed. Pearson’s chi-square (bilateral asymptotic significance) was used for the latter, with Fisher’s exact test (bilateral exact significance) for qualitative and dichotomized quantitative variables. The confidence interval was set at 95%. The SPSS program (version v. 23) was used for data processing.

The study was carried out in accordance with the ethical principles of the Declaration of Helsinki. All subjects consented in participate in the study.

## Results

A total of 1783 correctly completed surveys were obtained, out of the 2500 planned (1500 in addiction settings and 1000 in mental health settings). Of these, 322 came from mental health network resources and the rest from addictive disorder care resources. All subjects in the sample identified as either male or female (73.6% as men/male).

The data obtained show a significant concurrence of SUD and OMD diagnoses (in more than 2/3 of the patients). A high prevalence of OMD was found in those patients receiving treatment for their SUD (71%), and also of diagnoses of any SUD (68.9%) in people receiving treatment for diagnoses of OMD. Also were found significant relationships between addiction to certain substances and specific mental disorders: personality disorders with all SUDs; psychotic disorders with cannabis use disorder, but not cocaine use disorder; affective disorders with cocaine use disorder, and anxiety disorders with cannabis use disorder. The results are summarized in the tables below (Tables 1, 2, 3, 4, 5, 6, and 7).

**Table 1:** Sociodemographic and clinical (somatic) characteristics of the sample

	TOTAL *N*= 1783	MENTAL H. *n*=322	ADDICTIONS *n*=1461	Values of *F*; *x*^2^ , *p*
Gender (male)	1310 (73.6%)	216 (67.3%)	1094 (75%)	***7.67***; ***0.005***
Age (years) *	47.54 ± 1.38	48.30 ± 12.17	43.11 ± 11.21	***1.79***; ***0.04***
Living arrangements				
*Own family*	587 (33.9 %)	34.5 %	25.5 %	1.98; 0.07
*Alone*	462 (26 %)	27.3 %	32.5 %	1.33; 0.09
*With other people*	734 (40.1%)	38.2 %	42 %	1.05; 0.53
Employment status				
*Active or retired*	591 (29.3%)	20.8%	31.2%	***2.38***; ***<.01***
*Not working*	1192 (70.7%)	79.2%	68.8 %	***2.21***; ***<.009***
Marital status: single	908 (50.9%)	168 (52.2%)	740 (50.7%)	1.51; 0.624
HCV	292 (16.4%)	27 (8.4%)	265 (18.1%)	***4.97***; ***<.0001***
HCB	65 (3.6%)	7 (2.2%)	58 (4%)	1.05; 0.120
HIV	89 (5%)	5 (1.6%)	84 (5.7%)	***8.12***; ***0.002***
Neurological disease	71 (4%)	15 (4.7%)	56 (3.8%)	0.07; 0.493

*Mean, standard deviation

*p* values in bold italic are shown in the second place

**Table 2:** Actual diagnosis of substance use disorder (total, mental health network and addiction network [*N* (%)]

	TOTAL *N*= 1782	MENTAL H *n*= 322	ADDICTIONS *n*= 1461	Values *X*^2^; *p*
Any		222 (68.9%)	1396 (96.9%)*	***7.21***; ***0 .001***
Any (except tobacco)		142 (44.1%)	1328 (90.8%)	***8.68***; ***0 .002***
*Substance use disorder*				
Alcohol	744 (41.7%)	80 (24.8%)	664 (45.4%)	***18.32***; ***<.0001***
Opioids	370 (20.8%)	12 (3.7%)	358 (24.5%)	***28.93***; ***<.0001***
Cannabis	416 (23.3%)	41 (12.7%)	375 (25.7%)	***13.11***; ***<.0001***
Sedatives/hypnotics	116 (6.5%)	10 (3.1%)	106 (7.3%)	***15.07***; ***<.0001***
Cocaine	521 (29.2%)	33 (10.2%)	488 (33.4%)	***18.81***; ***<.0001***
Stimulants (Amphet.)	45 (2.5%)	12 (3.7%)	33 (2.3%)	1.02; 0.128
Hallucinogens	5 (0.3%)	0	5 (0.3%)	0.96; 0.293
Tobacco	643 (36.1%)	142 (44.1%)	501 (34.3%)	***8.98***; ***0.001***
Multiple drugs	36 (2%)	6 (1.9%)	30 (2.1%)	1.91; 0.826

*Rest not specified or not answered

*p* values in bold italic are shown in the second place

**Table 3:** Diagnosis of another mental disorder (not F10–19) (total, mental health network and addiction network [*N* (%)]

	TOTAL *N*= 1783	MENTAL H *n*= 322	ADDICTIONS *n*= 1461	Values *X*^2^; *p*
Any		297 (92.2%) *	1037 (71.0%)	***10.06***; ***<.0001***
Psychotic (f 20–29)	315 (17.7%)	113 (35.1%)	202 (13.8%)	***14.02***; ***<.0001***
Affective (f 30–39)	580 (32.5%)	108 (33.5%)	472 (32.3%)	0.06; 0.669
Anxiety (f 40–49)	296 (16.6%)	43 (13.4%)	253 (17.3%)	1.21; 0.084
Eat and sleep (f 50–59)	70 (3.9%)	10 (3.1%)	60 (4.1%)	0.09; 0.402
Personality(f 60–69)	459 (25.7%)	71 (22%)	388 (26.6%)	1.16; 0.094
Others (f 00–09, 70–99)	75 (4.2%)	7 (2.1%)	68 (4.7%)	***3.08***; ***0.042***

*Rest not specified or not answered

*p* values in bold italic are shown in the second place

**Table 4:** Substance use disorder and other mental disorder (*p* values)

*N*=1783	ALCOHOL	OPIOIDS	CANNABIS	SEDATIVES	COCAINE	OTH.EST.	OTH DRGS.	TOBACCO
ORGANIC	0.468	0.716	0.435	0.803	0.090	**0.020**	0.821	0.216
PSYCHOTIC	**0.012**	0.188	**0.000**	0.594	0.739	**0.000**	0.866	**0.000**
AFFECTIVE	**0.001**	0.112	0.276	0.079	**0.000**	0.079	0.562	0.161
ANXIETY	0.633	0.093	**0.006**	**0.013**	0.693	0.736	0.880	0.424
EAT & SLEEP	0.638	0.609	0.170	0.541	**0.046**	0.164	0.100	**0.044**
PERSONALITY	**0.033**	**0.000**	**0.000**	**0.000**	**0.000**	**0.031**	0.757	**0.000**
OTHER	0.597	0.079	**0.036**	0.690	**0.019**	0.129	0.831	**0.009**

Grades of freedom=1; *χ*^2^ Pearson’s values between 0.03 and 20.66

*p* values in bold are shown in all cases

**Table 5:** Prescribed pharmacological treatments

*N*=1783	TOTAL *n*= 1783	MENTAL H *n*= 322	ADDICTIONS *n*= 1461	Values *X*^2^; *p*
*For SUD (any)*	954 (53.5%)	96 (17.4%)	858 (65.4%)	***26.16***; ***<.00001***
Disulfiram/cyanamide	183 (10.3 %)	42 (13 %)	141 (9.7 %)	3.08; 0.069
Opioid agonists	423 (28.1%)	12 (3.7%)	411 (31.3%)	***31.12***; ***<.00001***
Opioid antagonists	57 (3.7%)	4 (1.9%)	53(3.9%)	1.05; 0.121
*For OMD (any)*	1329 (74.5%)	284 (88.2%)	1045 (71.5%)	***9.11***; ***<.0001***
Antipsychotics	599 (33.6%)	171 (52.1%)	428 (29.3%)	***13.23***; ***<.0001***
Mood stabilizers	294 (22%)	69 (23.2%)	225 (21.7%)	0.07; 0.574
Antidepressants	714 (53.5%)	129 (43.4%)	585 (56.4%)	***10.54***; ***<.0001***
*Anxiolytics*	779 (43.7%)	108 (36.4%)	671 (45.9%)	***11.96***; ***<.0001***

*SUD* substance use disorder; *OMD*, other mental disorder

*p* values in bold italic are shown in the second place

**Table 6:** Prescribed treatments by gender

*N*=1780*	Male (*n*=1310)	Female (*n*=470)	Value *χ*^2^; *p*
Opioid agonists	367 (28.1%)	54 (11.5%)	***13.95***; ***<.001***
Opioid antagonists	35 (2.7%)	22 (4.7%)	***1.88***; ***0.034***
Disulfiram/cyanamide	130 (9.9%)	53 (11.3%)	0.407
Anxiolytics/Hypnotics	540 (41.2%)	239 (50.9%)	***13.28***; ***<.001***
Mood stabilizers	228(18.9%)	66 (14.9%)	0.093
Antidepressants	457 (38.3%)	257 (54.7%)	***21.16***; ***<.0001***
Antipsychotics	452 (36.5%)	147 (31.3%)	0.059

*In 3 subjects, some data were not available

*p* values in bold italic are shown in the second place

**Table 7:** Comorbidity and gender

*N*=1780*	Male (*n*=1310)	Female (*n*=470)	Value *χ*^2^; *p*
SUBSTANCE USE DISORDER			
Alcohol	526 (40.1%)	218 (46.4%)	2.81; 0.08
Tobacco	486 (37.1%)	155 (33%)	0.110
Opioids	296 (22.6%)	74 (15.7%)	***9.03***; ***0.005***
Cannabis	329 (25.1%)	85 (18.1%)	***10.73***; ***0.002***
Cocaine	417 (31.8%)	104 (22.1%)	***17.5***; ***<.000***
Anxiolytics/hypnotics *non prescribed*	90 (6.9%)	26 (5.5%)	0.313
Others	72 (5.5%)	14 (3%)	0.068
OTHER MENTAL DISORDER			
F 0–9. Dementias	12 (0.9%)	6 (1.3%)	***13.11***; ***<.001***
F 20–29. Psychotic disorders	258 (18.9%)	57 (12.3%)	***9.82***; ***0.002***
F 30–39. Affective disorders	365 (29.3%)	215 (45.7%)	***14.51***; ***<.001***
F 40–49. Anxiety disorders	189 (15.2%)	107 (23.6%)	***12.44***; ***<.001***
F 50–59. Eating and sleep disorders	43 (3.4%)	37 (7.9%)	***21.92***; ***<.000***
F 60–69. Personality disorders	339 (26.2%)	119 (26%)	0.703
F 70–99. Other disorders	42 (3.3%)	12 (2.6%)	***3.82***; ***0.03***

Grades of freedom between 1 and 6; *x*^2^ values between 0.24 and 110.46; *in 3 subjects, some data were not available

*p* values in bold italic are shown in the second place

### Substance use disorder diagnoses

In the mental health network, 68.9% of patients had a current diagnosis of SUD. The use disorders (UD) of alcohol, tobacco, cocaine, and cannabis stand out in the total. In the addiction network, alcohol, tobacco, and cocaine UDs were the most common, while in the mental health network, these were tobacco, alcohol, and cannabis UDs. Furthermore, and not specified in Table 2, there was a significant co-occurrence of several current diagnoses for substance use (36.1%), the most frequent being those tobacco and alcohol UDs (10.6%) and cocaine and alcohol UDs (5.3%). The SUDs, both in the mental health network and in the addictions network, are shown in Table 2.

### Diagnoses of other mental disorders

There was a high prevalence of comorbid diagnoses in the addiction network, especially involving affective and personality disorders; in the mental health network, psychotic and affective disorders were the most commonly diagnosed. A significant difference in diagnosis between both networks was found in regard to psychotic disorders. The diagnoses of mental disorders not due to substance use (not F10–19, ICD-10) in both treatment networks are summarized in Table 3. Furthermore, and not specified in Table 3, there was a significant co-occurrence of several OMD (36.1%), the most frequent being personality disorder with affective disorder (17.7%) and with anxiety disorder (12.6%).

### Relationships between SUDs and other mental disorders

The relationships found between the different SUDs and other mental disorder diagnoses, grouped by major syndromes, are shown in Table 4.

The most relevant significant relationships found between the UD of specific substances and the different specific mental disorders were as follows: alcohol UD with affective disorder; opioid UD with personality disorder; cannabinoid UD with psychotic disorder, personality disorder, and anxiety disorder; UD sedatives with personality disorder, anxiety disorder, and sleep disorder; cocaine UD with affective disorder and personality disorder, not related to psychotic disorders; stimulant UD (amphetamines) with psychotic disorder and personality disorder; and tobacco UD with psychotic disorder and personality disorder.

With regard to non-SUD mental disorders, the most significant relationships were: psychotic disorders with cannabis, other stimulants and tobacco UDs, and to a lesser extent with alcohol UD; affective disorder especially with cocaine UD and less with alcohol UD; anxiety disorder with cannabis UD and less significantly with sedative UD. Personality disorders are related to all UDs, but especially opioid UD, cannabis UD, sedative UD, cocaine UD, and tobacco UD.

### Prescribed treatments

Disulfiram or cyanamide were relatively little used in both networks; opiate antagonists were hardly ever used in either health care network; the use of opiate agonists for the treatment of opioid dependence occurred almost exclusively in the addiction network; a low percentage of patients were treated for their SUDs in the mental health network, despite their high prevalence. Psychotropic drugs were used similarly for their treatment in both networks, with considerable use of antipsychotics and frequent prescription of antidepressants and mood stabilizers. There was a very high percentage of patients who were prescribed anxiolytics/hypnotics in both treatment networks. The pharmacological treatments prescribed in the two care networks are summarized in Table 5.

Proportionally, women had fewer treatments with agonists and more with antagonists. They had more treatments with anxiolytics and antidepressants. Disulfiram/cyanamide, mood stabilizers, and antipsychotics were prescribed to the same extent as in men. Differences in pharmacological prescriptions by gender are reflected in Table 6.

### Comorbidity and gender

Women lived in a higher percentage with their own family (43% vs. 29.2%; *x*^2^ value: 23.11, *p* < 001) and less with a family of origin than men (23.4% vs. 36.8%; *x*^2^ value: 26.12, *p* < 0.001), and that they were pensioners (retired) in a lower percentage (21.7% vs. 27.4%; *x*^2^ value: 1.81, *p*= 0.029), with no significant differences in active working compared to men. Hepatitis B and C and HIV were more frequent in men than in women. Women were less frequently diagnosed with cannabis, opioid, and, especially, cocaine use disorders. And they had fewer psychotic disorders and more affective, anxiety, sleep, and eating disorders, with the rest being the same, including personality disorders. The findings differentiated by gender are specified in Table 7.

## Discussion

### Sociodemographic and clinical profile and substance use

The sociodemographic characteristics of our sample did not differ greatly from that already known from different studies in Spain (Fernández-Miranda et al. 2001; Roncero et al. 2011; Arias et al. 2013; Pereiro et al. 2013). The most common SUDs in both networks were alcohol, tobacco, cocaine, and cannabis disorders, which largely correspond to what is known about the populations in treatment in Spain (Gual 2007; Roncero et al. 2011; Pereiro et al. 2013; Pascual-Pastor et al. 2017). It is remarkable that in the mental health network, 68.9% of patients had a current SUD diagnosis (59% excluding tobacco).

With regard to diagnoses for other mental disorders, the fundamental finding is that 71% of patients in the addiction network were diagnosed with a mental disorder other than addiction, a very high prevalence. The frequent co-occurrence of SUDs and affective (32.3%), personality (26.6%), anxiety (17.3%), and psychotic disorders (13.8%) is consistent with what has generally been shown by some previous studies in Spain (Fernández-Miranda 2002; Gual 2007; Roncero et al. 2011; Pereiro et al. 2013).

### Treatment of co-occurring diagnoses

Since the 1980s, there have been two networks in Spain to treat a single patient with an addictive disorder and other mental disorders; this has continued in many cases to the present day, with a very differentiated treatment network persisting in some autonomous communities for patients with addictions. As a result, the comorbidity is approached in parallel or sequential care, leading to deficits which increase morbidity and mortality and the abandonment of treatment (Roncero et al. 2011; Pascual-Pastor et al. 2017), as is the case in other countries (Mueser et al. 2003; Mangrum et al. 2006; Torrens et al. 2015; Priester et al. 2016). It is important to highlight the lack of integrated treatment as an approach strategy, although this model has been shown to be more effective than approaching each disorder with separate treatment plans (Drake et al. 1998; Mueser et al. 2003; Donald et al. 2005; Torrens et al. 2012; Arias-Horcajadas et al. 2020; Spivak et al. 2020). A number of studies assessing both psychosocial and pharmacological interventions have shown promise and could guide clinical practice (Iqbal et al. 2019; Murthy et al. 2019; Arias-Horcajadas et al. 2020). Ideally, they should be of high intensity and based on established SUD therapies (Tiet and Mausbach 2007; Iqbal et al. 2019; Hunt et al. 2019; Arias-Horcajadas et al. 2020; Spivak et al. 2020). The treatments used in patients with dual disorders are, in general terms, rather similar to those used when a single pathology is present.

In general terms, it is considered that treatments which are effective for mental disorders are also effective in dual diagnosis patients; and that treatments indicated for substance use disorders are also suitable for psychiatric patients with SUDs (Sáiz Martínez et al. 2014; Tirado Muñoz et al. 2017; Crockford and Addington 2017; Arias-Horcajadas et al. 2020). But there are some particularities that should be noted. Treatment with non-SSRI antidepressants should be considered for patients with depression and SUD: adding a more dopaminergic and noradrenergic profile or mixed mechanisms of action appears to be more effective (Tirado Muñoz et al. 2017). SSRIs are considered first-line therapy in the treatment of dual anxiety while benzodiazepines should be avoided (Sáiz Martínez et al. 2014). Concerning antipsychotics, there is no evidence of any differential benefit for one antipsychotic over another for people with psychosis and coexisting SUD (Crockford and Addington 2017). One exception is clozapine, which has been shown to have an impact on the reduction in craving for cannabis, and to have an edge over other antipsychotics in people with schizophrenia and SUD (Murthy et al. 2019). Given the poor adherence to medication, LAIs as a first-line option are recommended, especially risperidone and aripiprazole (Azorin et al. 2016). Although no psychopharmaceutical treatment is contraindicated ( Azorin et al. 2016; Iqbal et al. 2019; Murthy et al. 2019; Arias-Horcajadas et al. 2020), benzodiazepines are not recommended (Sáiz Martínez et al. 2014).

In our research, a low percentage of patients were treated for their SUDs in the mental health network, despite their high prevalence. Another finding is that disulfiram or cyanamide was relatively little used in both mental health and addiction networks. The reason for 370 people with opioid use disorder but 423 patients on opioid agonists is due to the fact that a considerable number of patients undergoing opioid maintenance for many years currently have cocaine or other stimulants UD as their main SUD. Although psychotropic drugs were used similarly for their treatment in both settings, there must be highlight that there was a very high percentage of patients (50%) who were prescribed anxiolytics/hypnotics in both treatment networks, despite their clear contraindication and their risk of abuse (Sáiz Martínez et al. 2014; Guardia-Serecigni and Flórez-Menéndez 2018). This finding about the treatment of patients with dual diagnosis, regardless of where they are treated, should be considered by professionals, in particular when its specific indication is clearly established, as well as the duration of its prescription in general terms (Smith and Randall 2012; Sáiz Martínez et al. 2014).

### Gender perspective

The fact that are there less women in SUDs treatment than men, that women live in a higher percentage with their own family and less with a family of origin than men, and that they are pensioners in a lower percentage coincides with the profiles usually shown both in one network and in addiction treatment network (Fernández-Miranda et al. 2001; Gual 2007; Arias et al. 2013; Pereiro et al. 2013; Pascual-Pastor et al. 2017). Hepatitis B and C, and HIV, are more frequent in men than in women, as our research shows; this fact could be a consequence of the more habitual risk behaviors in men, which has also been pointed out by different authors (Fernández-Miranda et al. 2001; Torrens et al. 2012; Pereiro et al. 2013).

Regarding the current SUD, in our research, women have less diagnosis of opioids, cannabis, and cocaine UDs. The lower relationship with the problematic use of opioids and cannabis by women is a fact that is not easy to explain, and for which there are studies with inconclusive findings (Fernández-Miranda et al. 2001, 2019; Gual 2007; Cuenca-Royo et al. 2013; Pereiro et al. 2013; Pascual-Pastor et al. 2017; Ochoa-Mangado et al. 2018). As for other mental disorders diagnosed at the time of the survey, women have fewer psychotic disorders and more affective, anxiety, sleep, and eating disorders, with the rest being the same, including personality disorders. Although the higher prevalence of affective (and also anxiety) disorders in women is a common finding (Conner et al. 2009; Sánchez-Peña et al. 2012; Miquel et al. 2013; García-Carretero et al. 2017; Guardia-Serecigni and Flórez-Menéndez 2018; Palma-Álvarez et al. 2019), it is not so that PDs are just as frequent in men than in women (Fernández-Miranda 2002; Sánchez-Peña et al. 2012; Miquel et al. 2013; Torrens et al. 2015), as our study shows.

Depression may be an important factor during the approaching of SUDs because it could be related to treatment general outcomes and compliance (Zilberman et al. 2007; Gjestad et al. 2011; Merrill et al. 2014; Abulseoud et al. 2013; Luminet et al. 2016; Thibaut 2018); and also with suicidal behavior (Darvishi et al. 2015; Rodríguez-Cintas et al. 2018). Moreover, women with SUDs and OMDs are related to a greater craving than men (which can lead to a relapse) (Zilberman et al. 2007; Gjestad et al. 2011; Merrill et al. 2014; Luminet et al. 2016; Robles-Martínez et al. 2018). Besides, differences in relation to the higher presence of psychotic symptoms in men than in women have been suggested (Jordaan and Emsley 2014), as in our research. The high prevalence found in our investigation between affective disorders and SUDs has to be highlighted.

### Treatments by type of care network and gender

Our study shows how treatments for OMDs are very common in addiction treatment networks, reflecting the important awareness within them of psychiatric comorbidity and the need to treat it. It is, however, remarkable that, despite their high prevalence, the treatment of SUDs in patients treated in mental health networks is lower than might be expected (Mangrum et al. 2006; Grau-López et al. 2014). This could point to precisely the opposite of the addiction network: insufficient concern and attention to SUDs in people initially treated for OMD (in less than 20% of them). On the other hand, the co-occurrence of OMDs is treated with similar medications in the addictions network than in the mental health network (Fernández-Miranda et al. 2001; Roncero et al. 2011; Arias et al. 2013; Pascual-Pastor et al. 2017).

Regarding the gender approach in the treatment of comorbidity, it has been described that there are gender differences in the access to treatment resources specialized between men and women, due to several factors, such as social stigma and lack of specialized resources (Miquel et al. 2013; Palma-Álvarez et al. 2019). Some studies show that better results are obtained in terms of attracting women with SUD for treatment and in higher rates of adherence when differentiated treatments for women are used (Coughey et al. 1998; Ashley et al. 2003; Abulseoud et al. 2013)). Women are at greater risk for psychiatric comorbidity (Torrens et al. 2011; Fonseca et al. 2021), as our study shows (with higher prevalence of all OMDs in women, except psychotic disorders, higher in men, and with no differences regarding PDs). This must be considered when identifying pharmacological and/or behavioral interventional strategies because it may differ from those most beneficial for men. For these reasons, it is important to guarantee access to the appropriate treatment of females who have addictive and other mental disorders.

In our research, although the prevalence of opioid use disorder is significantly lower among women, they receive proportionally more treatment with antagonists than with agonists compared to men (4.7/11.5% vs. 2.7/20.4%), which may show a bias regarding inclusion in demonstrated useful programs that are less accepting women (Miquel et al. 2013; Fernández-Miranda et al. 2019; Arias-Horcajadas et al. 2020). They have more treatments with anxiolytics and antidepressants, which is not surprising knowing the use of these drugs and their prescription for anxiety and depressive disorders, that are more prevalent in women in the general population, in comorbid patients (Conner et al. 2009; Miquel et al. 2013; García-Carretero et al. 2017; Thibaut 2018; Arias-Horcajadas et al. 2020), and in our research. Disulfiram/cyanamide, mood stabilizers, and antipsychotics are prescribed to them to the same extent as men, which reflects the inexistence of any bias in the prescription of these families of psychoactive drugs (Sáiz Martínez et al. 2014; Tirado Muñoz et al. 2017; Guardia-Serecigni and Flórez-Menéndez 2018; Vitali et al. 2018).

### Strengths and limitations of the study

This research is the first to be carried out in Spain with an objective clearly national in scope, and also reflecting the diversity of healthcare networks, with a sample of significant size. A descriptive approach and an inclusive definition of comorbidity were used. Given the above, it can be considered representative of people with diagnoses of SUD and on current OMDs in treatment throughout Spain. In short, the main strengths of this study are the size of the sample, the national scope, and the variety of treatment settings to avoid biases as far as possible, thereby ensuring an acceptable level of representativeness. In this way, specific aspects of women regarding prevalence and treatments could be studied. Although this study was carried out to prospect gender differences and changes, sadly the percentage of men is quite higher than that of women, but also since this is a clinical population and as have been mentioned accessing services for women is lower.

A possible limitation of this study is that the inclusion of cases was not carried out in a uniform manner and could have resulted in bias in sample collection, especially given the fact that patients treated in the mental health network are less represented than those with addictions. This lower response in the mental health network to the surveys could reflect less awareness of the problem in this network. A bias in the patient sample may also result not only from the low profile of the mental health network but also from the heterogeneity of the care structures themselves throughout the country. Nevertheless, it reflects the reality of care for SUDs and OMDs in Spain. A further possible limitation could also be the randomization method, which was chosen for its ease of application.

Additionally, instead of validated questionnaires, the one used was constructed ad hoc, easy to fill out, and collected the most relevant variables for the objective of the study. It should be clarified that if only pharmacological treatments were chosen for the study, this was due to the methodological difficulties in registering the types of psychological treatments, as they are not registered with the same precision as medicinal ones. Finally, it should be noted that comparability with other studies is limited since there are hardly any studies of such a general nature in our setting.

## Conclusions

Given the characteristics of its design and notwithstanding its limitations, this research can provide indicative and valuable preliminary information on the prevalence of SUD and OMD co-occurrence, and medication provided, in both the mental health and the addiction networks throughout Spain, and on the differences by gender on both issues. In general, the data obtained show a significant co-occurrence of diagnoses (in more than 60% of patients), with some differences between women and men: women are at greater risk for psychiatric comorbidity (with higher prevalence of all OMDs, except psychotic disorders). This must be considered when identifying interventional strategies because it may differ from those most beneficial for men. We found also low intervention in SUDs in mental health settings, common treatments for OMDs in addiction treatment networks, a high prescription of benzodiazepines in both networks, and certain biases related to gender related to medications prescribed and lower access to treatment services for women with this comorbidity

This study provides preliminary information on the coexistence in routine clinical practice of addictive disorders and other mental disorders in Spain, and on the treatment provided, reflecting the comorbidity in normal conditions of clinical practice and the possible distortions in how this is treated in women. In fact, it shows some differences in prevalence and clinical characteristic, and especially in treatment approaches between women and men. Thus, should be useful to adapt the treatment response with greater precision, and with a gender perspective.

## Appendix. Ad hoc questionnaire used

### Resource

Specific Addiction care ambulatory center

Ambulatory Mental Health Unit

Hospital Mental Health Rehabilitation Unit

Hospital Addiction Rehabilitation Unit

Day hospital for people with addiction

Day hospital for people with psychiatric disorders

Prisons

City

### Sociodemographic

Gender

*Woman*

*Man*

*Other:*

Year of birth

Marital status

*Married*

*Bachelor*

*Widower*

*Separate*

Living arrangements

*Alone*

*Origin family*

*Own family*

*Institution*

Employment Status

*Active*

*Not working*

*Pensioner*

*Retired*

*Other*

### Somatic diseases

Hepatitis C

Hepatitis B

HIV

Neurological disease

Other:

### Substances consumed in the last month

Tobacco

Alcohol

Cannabis

Cocaine

Stimulants (e.g. amphetamine)

Heroin

Prescription opioids

Other:

### Substances you have used but not in the last month

Tobacco

Alcohol

Cannabis

Cocaine

Stimulants (e.g. amphetamines)

Heroin

Prescription opioids

Other:

### Substance Use Disorder in the last twelve months

F10. SUD due to alcohol consumption

F11. SUD due to Opioid Use

F12. SUD due to cannabinoid use

F13. SUD due to sedative or hypnotic use

F14. SUD due to cocaine use

F15. SUD due to the use of other stimulants (including caffeine)

F16. SUD due to hallucinogenic use

F17. SUD due to tobacco use

F18. SUD due to the consumption of volatile solvents

F19. SUD due to the use of multiple drugs or other psychotropic substances

### Substance use disorder more than 12 months ago

F10. SUD due to alcohol consumption

F11. SUD due to Opioid Use

F12. SUD due to cannabinoid use

F13. SUD due to sedative or hypnotic use

F14. SUD due to cocaine use

F15. SUD due to the use of other stimulants (including caffeine)

F16. SUD due to hallucinogenic use

F17. SUD due to tobacco use

F18. SUD due to the consumption of volatile solvents

F19. SUD due to the use of multiple drugs or other psychotropic substances

### Other mental disorders

F00. Dementia

F09. Organic mental disorder without specification

F20. Schizophrenia

F21. Schizotypic disorder

F22. Persistent Delusions

F23. Acute psychotic disorder

F25. Schizoaffective

F31. Bipolar

F32. Depressive episodes

F33. Recurrent depressive disorder

F 34. Persistent (affective) mood disorder

F39. Affective disorder without specification

F40. Phobic anxiety disorder

F41. Other Anxiety disorder

F42. Obsessive-compulsive disorder

F 43. Severe stress reactions and adaptation

F44. Dissociative disorders

F45. Somatomorph disorder

F50. Eating behavior disorder

F51. Non-organic sleep disorder

F52. Non-organic sexual dysfunction

F55. Substance abuse that does not produce dependence

F60. Personality-specific disorder

F63. Habits and impulse control disorder

F64. Sexual Identity disorder

F69. Personality disorder without specification

F70-F79. Mental retardation

F84. Development disorder

F90. Hyperkinetic disorder

F99. Unspecified disorder

### Current treatment

Antipsychotics

Opioid agonists

Opioid antagonists

Disulfiram

Euthymizants

Antidepressants

Anxiolytics

Other
